# Associations between Health Literacy and Sociodemographic Factors: A Cross-Sectional Study in Malaysia Utilising the HLS-M-Q18

**DOI:** 10.3390/ijerph18094860

**Published:** 2021-05-02

**Authors:** Arina Anis Azlan, Mohammad Rezal Hamzah, Jen Sern Tham, Suffian Hadi Ayub, Abdul Latiff Ahmad, Emma Mohamad

**Affiliations:** 1 Centre for Research in Media and Communication, Faculty of Social Sciences and Humanities, Universiti Kebangsaan Malaysia, Bangi 43600, Selangor, Malaysia; arina@ukm.edu.my (A.A.A.); alba@ukm.edu.my (A.L.A.); 2 HEALTHCOMM UKM x UNICEF C4D Centre, Faculty of Social Sciences and Humanities, Universiti Kebangsaan Malaysia, Bangi 43600, Selangor, Malaysia; 3 Centre of Excellence for Social Innovations and Sustainability, Faculty of Applied and Human Sciences, Universiti Malaysia Perlis, Kangar 01000, Perlis, Malaysia; rezal@unimap.edu.my; 4 Department of Communication, Faculty of Modern Languages and Communication, Universiti Putra Malaysia, Seri Kembangan 43400, Selangor, Malaysia; jstham@upm.edu.my; 5 Faculty of Communication and Media Studies, Universiti Teknologi MARA, Shah Alam 40450, Selangor, Malaysia; suffianhadi@uitm.edu.my

**Keywords:** health literacy, HLS-M-Q18, sociodemographic associations, health communication

## Abstract

Health literacy is progressively seen as an indicator to describe a nation’s health status. To improve health literacy, countries need to address health inequalities by examining different social demographic factors across the population. This assessment is crucial to identify and evaluate the strengths and limitations of a country in addressing health issues. By addressing these health inequalities, a country would be better informed to take necessary steps to improve the nation’s health literacy. This study examines health literacy levels in Malaysia and analyses socio-demographic factors that are associated with health literacy. A cross-sectional survey was carried out using the HLS-M-Q18 instrument, which was validated for the Malaysian population. Multi-stage random sampling strategy was used in this study, utilising several sampling techniques including quota sampling, cluster sampling, and simple random sampling to allow random data collection. A total of 855 respondents were sampled. Our results showed that there were significant associations between health literacy and age, health status, and health problems. Our findings also suggest that lower health literacy levels were associated with the younger generation. This study’s findings have provided baseline data on Malaysians’ health literacy and provide evidence showing potential areas of intervention.

## 1. Introduction

Worldwide interest in studying health literacy is increasing as health promoters and practitioners recognise its significance in reducing illness [1] and improving quality of life [2]. The benefits of health literacy extend beyond individual health care to include effective disease prevention in society, as well as improving health promotion in general. Health literacy is a concept that extends beyond health education. It addresses social and environmental factors that influence individual ability to engage with health information, to make informed decisions, and to utilise health services to benefit them and their surroundings.

Studies have emphasised a variety of health literacy benefits to society [3] and reported risks of populations with low health literacy [4]. Higher health literacy has also been associated with positive health outcomes [5], healthy behaviours [5,6], and lower health costs [7]. It is plausible to expect that the way forward to realise better global health management is to improve society’s health literacy levels.

As awareness of the importance of health literacy increases, more instruments are being developed to accurately measure health literacy rates among general populations, as well as in specific groups. A 2017 systematic review revealed 36 instruments used to measure health literacy [8], while another systematic review in 2018 reported 29 health literacy instruments used on children and adolescents [9]. In Malaysia, several tools have been utilised to measure health literacy [10], the most recent being the HLS-M-Q18, consisting of 18 items to accommodate for the Malaysian National Health Morbidity Survey in 2019 [11].

Even so, the measurement of Malaysian health literacy at the national level is still in its infancy. Previous studies have focused on measuring health literacy among specific groups or in specific illnesses [10]. The results of these studies have shown that the health literacy level in Malaysia is limited to moderate. However, because the studies were conducted in smaller contexts, their findings are diverse. For instance, one study on overweight housewives found significant associations between older age, low education levels, low income, and adequate health literacy [12]. Another study on the acquisition of health information found that older individuals, those living in rural areas, individuals suffering from chronic disease, and those with a history of serious family illnesses were less likely to acquire poor health information [13].

Globally, studies on health literacy also vary in population subgroups and illnesses. The findings of a comprehensive examination of health literacy in Europe indicate that subgroups of the population that are older in age, have lower income, and have lower education levels had higher proportions of individuals with low health literacy [14]. A study conducted in six Asian countries also found that higher levels of education and social status were associated with higher health literacy [15]. The findings of these studies have aided health authorities in planning intervention programmes to increase health literacy among those who are lacking it. The varying results of the health literacy studies conducted in Malaysia warrant a closer examination to determine the health literacy levels of the general Malaysian population and the differences within its subgroups.

The objectives of this study are to (1) measure society’s health literacy and (2) observe socio-demographic factors that are associated with health literacy in Malaysia. In order to improve health inequalities in the community, the assessment of individual health literacy is crucial to identify and evaluate the strengths and limitations in addressing health issues in a diverse society [16]. In order for health care providers and policy makers to respond efficiently, they need to understand the diverse factors that affect health literacy before facilitating access to health information, providing services, and devising health intervention that do not discriminate against health literacy limitations [17].

## 2. Materials and Methods

### 2.1. Study Design

A nationally representative cross-sectional survey was employed to address the research objectives. The survey was administered by well-trained enumerators who were also staff working for the Ministry of Health Malaysia. Three states were selected (Selangor, Kuala Lumpur, and Sarawak) to represent the distribution of multiple ethnicities, as well as the distribution of urban and rural areas. The selection of areas was made based on referral and advice from the District of Jurisdiction Malaysia, the Rural Master Plan Malaysia, and previous literature [18].

### 2.2. Ethical Approval

The National Medical Ethics Committee Malaysia under the Ministry of Health Malaysia approved our study protocol, procedures, information sheet, and consent statement (NMRR-18-1320/41882). All the respondents were above 18 years old, and therefore no minors were involved. All the respondents also signed a written consent form clearly stating their rights, and the nature of their participation in the study before being asked to answer the questionnaire. The confidentiality of the information and the privacy of the respondents were protected throughout the study.

### 2.3. Recruitment Procedure

**Figure 1:**
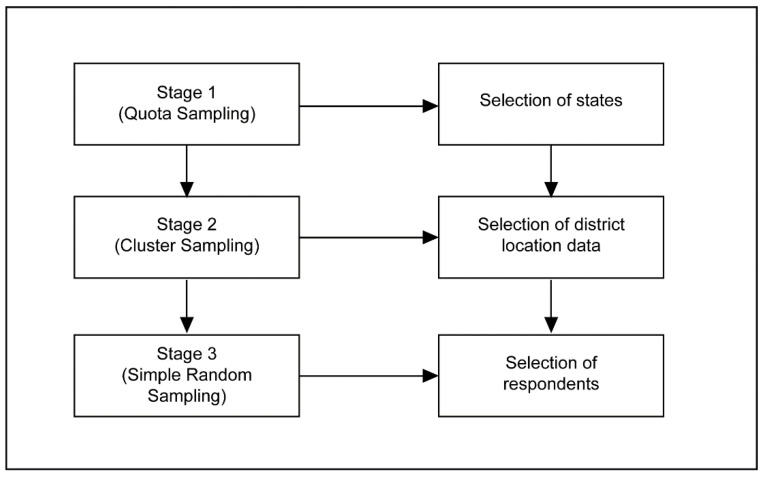
The multi-stage random sampling procedure.

Multi-stage random sampling was used in this study. In detail, there were three stages involved, utilising several sampling techniques (quota sampling, cluster sampling, and simple random sampling) to allow random data collection. The three stages are illustrated in Figure 1.

In stage 1, quota sampling based on ethnicities and urban or rural distribution was used to select three Malaysian states. Ethnic distribution should be a standard in sampling multiracial populations to ensure the inclusivity of the sample [19]. Malaysia consists of Peninsular Malaysia and Borneo. States from each were selected to represent the diverse ethnicities in Malaysia. For the purpose of urban and rural distribution, Kuala Lumpur and Sarawak were selected to represent the urban and rural areas, respectively. This is justified, as Kuala Lumpur has the highest urban population, while Sarawak has the highest rural population in Malaysia. In selecting the state of Sarawak, a more balanced representation of the minority ethnic groups could be obtained (i.e., Bumiputera). Selangor represents both the urban and rural areas and has a balanced ratio of ethnic group distribution.

In stage 2, cluster sampling was utilised to determine districts of choice. District sampling for Selangor was determined based on the demographic distribution list published by the Selangor Economic Development Unit, as well as by extant literature [20]. For selection of districts in Kuala Lumpur, researchers used data provided by the Department of Information, the Ministry of Communications, and Multimedia Malaysia; for Sarawak, the selection of districts was guided by data provided by the State Director of the Fire and Rescue Department. The definitions of rural and urban were determined by the National Department of Statistics and The Rural Master Plan, published by the Ministry of Rural Development Malaysia [21].

In stage 3, respondents were selected using a simple random sampling technique based on several criteria. Screening questions were developed to ensure the inclusion criteria were met (i.e., Malaysian, aged 18 and above, resident in the chosen state, makes their own health decisions). The respondent recruitment method used in this study mirrors the method and protocol criteria used by the Asian Health Literacy Consortium and those of previous literature [22]. If there were no eligible respondents in the household who met the selection criteria, household members were thanked for their time and the enumerator then approached the next selected household. Only one respondent from any given household was interviewed, and the eldest household member was chosen if there was more than one household member who met the respondent selection criteria.

The researchers made the decision to prioritise an inclusive Malaysian sample based on ethnicity and urban/rural strata due to constraints in resources. This was to ensure that the smaller groups were adequately represented in the sample. The list of states, ethnicities, and urban/rural distribution required for this study are presented in Table 1.

This cross-sectional survey was conducted between 25 June 2018 and 14 July 2018, and involved 18 enumerators. All the trained enumerators were working for the Ministry of Health Malaysia, wore the Ministry’s uniform, and presented their identity cards to avoid misunderstandings and to protect the interests of both researchers and respondents. Respondents took an average of 30–40 min to complete the questionnaire. The target sample size was 470, determined by identifying the smallest acceptable size of a demographic subgroup with a ±5% margin of error and a confidence level of 95% [23,24]. The enumerators went from household to household within the selected areas and provided the self-administered questionnaire to be answered. However, if respondents needed further clarification on the questionnaire, the enumerators would assist. A consent form was filled in and obtained from each respondent. A total of 866 complete responses with no missing data were obtained and analysed.

### 2.4. Study Instrument

The survey instrument was adopted from the HLS-M-Q18 short version of the health literacy questionnaire which was validated in a study [11]. The questionnaire contained three main sections: (1) demographics, which surveyed respondents’ socio-demographic information, including gender, age, race, marital status, and income; (2) personal health information; (3) an 18-item measure of health literacy. The questionnaire was constructed in the English and Malay languages. A backward-translation approach was used in translating the items from English to Malay, so as to ensure linguistic and conceptual equivalence [25]. Discrepancies between the two versions were rectified, and the equivalence of measures between all items was ensured through consultation with bilingual researchers.

Personal health information was measured by three items. First, respondents were asked to rate their health condition (self-rated) from “bad”, coded as “1”, to “good”, coded as “2”. The second item asked respondents to identify if they suffered from a long-term illness: “Do you have any long-term illness or health problems? Long-term illness means problems which have lasted, or you expect to last, 6 months or more”. Two answer options were provided (1 = yes, one or more than one, and 2 = no). The third item asked respondents to identify the frequency of their involvement in physical activities such as lifting and carrying heavy objects, hoeing, mopping the floor, and exercising (such as cycling, walking, or jogging) for at least 10 min in the past 7 days.

To measure respondents’ health literacy, 18 items were adopted from a validated Malaysian version of the HLS-EU-Q47 [11]. Respondents were asked to identify the level of difficulty, ranging from 1 = very difficult to 4 = very easy. An index was created based on the 18 items above (Table S1).

Demographic variables were controlled to reduce confounding effects. These variables included age, year of birth (1950 to 1965 for Baby Boomers, 1966 to 1976 for Generation X, 1977 to 1994 for Generation Y, and 1995 to 2012 for Generation Z), gender (0 = female, 1 = male), race (1 = Malay/Bumiputera, 2 = Chinese, 3 = Indian), marital status (1 = not married, 2 = married, 3 = separated/divorced, 4 = widowed), and monthly household income (1 = below RM3,000, including no income; 2 = RM3,001 to RM9,000; 3 = RM9,001 or more).

### 2.5. Statistical Analysis

For this study, the collected data were analysed using the Statistical Package for the Social Sciences (SPSS), version 26 (SPSS Inc., Chicago, IL, USA). Descriptive analysis focused on frequencies and percentages. Logistic regression tests using the Enter method were conducted to examine the relationships between control variables, personal health information, and health literacy. For this analysis, the levels of health literacy were re-coded to 0 = limited (inadequate and problematic) and 1 = adequate (sufficient and excellent). Odds ratios (OR), 95% confidence intervals (CI), and their corresponding *p* values are reported as indicators of the magnitude and statistical significance of associations.

## 3. Results

### Demographic Characteristics

A total of 866 respondents from different demographic segments and backgrounds participated in this study. The demographics were broadly representative of the Malaysian population, with slightly fewer male participants at 34.9%, and 65.1% female participants. Almost 70% of the study participants were from the younger generations (Z and Y). Table 2 shows the distribution of respondents according to selected demographics. The majority of the respondents were female, Malay, from generations Y and Z, and not married, and had low income levels.

Over 28% of the respondents perceived their general health as poor, but over 70% perceived their health status to be excellent or fairly good. Of the 866 respondents, 277 (17.8%) had inadequate health literacy, another 40.4% had marginal health literacy, 32.9% had adequate health literacy, and 9.1% had excellent or very good health literacy. On the average, the results of the study show that the younger generation (aged 33.1–33.8 years) was represented across all levels of health literacy.

Several socio-demographic characteristics were associated with health literacy level and are shown in Table 3. Characteristics associated with health literacy level included health status, health problems, and age. The logistic regression model was statistically significant, χ^2^ (4) = 49.285, *p* < 0.000. The model explained 7.6% (Nagelkerke R2) of the variance. The Hosmer and Lemeshow Test showed that the model was a good fit to the data as *p* = 0.954 (>0.05).

In terms of age, the findings revealed that there was a significant relationship between age and health literacy. Generation Y participants (aged 23–37) were less likely to be associated with adequate health literacy (OR 0.549, C.I = 0.319–0.946, *p* = 0.031 < *p* = 0.05). Respondents who had a self-perceived poor health status were less likely to be associated with adequate health literacy (OR 0.431, C.I = 0.301–0.618, *p* = 0.000 < *p* = 0.05), compared with those who rated their health as good. This indicates that, if the level of self-perceived poor health status increases, the odds of being associated with adequate health literacy will decrease. The association between health problems and the level of health literacy was statistically significant. Respondents who reported that they had “one or more than one” issue were nearly 1.5× more likely to be associated with adequate health literacy compared with those who had no disease (OR 1.447, C.I = 1.000–2.096, *p* = 0.05 < *p* = 0.05). The logistic regression results also showed that the other characteristics, such as gender, race, marital status, income, and daily exercise, remained not significantly associated with health literacy level.

## 4. Discussion

The results of our study indicate that Malaysians with one or more diseases were significantly more likely to have higher health literacy levels. The same pattern was observed in a study conducted among university students in Turkey [26] and in a previous study in Malaysia [13]; health literacy was significantly higher in those with chronic conditions. A possible explanation for this is that people with a diagnosis of long-term illness(es) were better acquainted with the healthcare system, health advice, and information. The MOH has targeted programmes among vulnerable populations, including free health screening, mobile health clinics, and community engagement programmes for health education. However, this raises concerns regarding the point at which people begin to build higher levels of health literacy. Familiarity with health information and services as a result of a long-term illness diagnosis may not benefit the individual in terms of disease prevention, early detection, and early treatment.

On the other hand, the results also showed that people with no long-term diseases were less likely to have adequate health literacy. This suggests that those with no long-term illnesses may be more complacent in acquiring health knowledge and healthy behaviours, while those with a long-term illness are more motivated to learn and engage in self-health management. Previous studies have found similar results; individuals who were active in maintaining and improving their health were those who had higher motivation to do so [27].

In terms of health status, our findings reveal that people who perceived themselves to have poor health were less likely to have adequate health literacy. This is consistent with extant literature indicating that those with low self-rated health tend to believe that it is due to insufficient information in managing their health. The study further suggested that this led to low confidence in navigating the healthcare system, thus affecting health literacy [28]. Another study found that individuals with poor or very poor self-assessment of health were more likely to have lower levels of health literacy [29]. It can be deduced that health literacy may be an important component in building individual self-efficacy for health management.

The present study also found that people between the ages of 23 and 27 were less likely to have adequate health literacy. Research conducted in Denmark found similar results, where lower health literacy was recorded among the younger population [30]. In another study, adults aged between 25 and 45 years were also found to have more difficulties with health literacy compared with older individuals [29]. This is worrying, considering the rampant health misinformation on the internet and its widespread use among the young generation. In previous studies, millennials were found to refer to online reviews prior to deciding on a physician for consultation [31]. With evidence that social media negatively contributes to the propagation of misinformation [32], this poses a threat to public health systems where the accuracy of health-related information is concerned. In Malaysia, studies on eHealth literacy are still in their infancy [33,34].

## 5. Limitations

A multi-stage sampling procedure was conducted to select the respondents in this survey. The sampling procedure prioritised ethnic group and urban/rural strata, important components in sampling multiracial populations to ensure inclusivity [19]. As a result, the gender and age distribution of the sample does not accurately reflect the current Malaysian population. The respondents of the study consisted of 65% women, while current Malaysian population estimates show that only 49% of the population is female. Similarly, 51% of the study sample was aged between 25 and 42 years of age. Malaysian population estimates show that only 32.9% of Malaysians are between the ages of 25 and 42 years.

The instrument utilised in this survey was the HLS-M-Q18, the shortened version of HLS-EU-Q47 tested for the Malaysian population. While this is beneficial for the overall assessment of health literacy, this has limitations in that the three health literacy domains were not measured independently. Therefore, the results of this study must be interpreted with caution.

## 6. Conclusions

Prior to the development of the Malaysian adaptation of the HLS-EU-Q47, health literacy in Malaysia was assessed utilising different instruments ranging from the Newest Vital Signs [35] to tools addressing specific disease literacy such as in dentistry [36,37] and in mental health [38]. The HLS-M-Q18 has enabled the measurement of health literacy in line with current global standards. Our study found that self-perceived health status and health problems were associated with health literacy levels. Markedly, lower health literacy levels were found to be associated with the younger generation. This is especially concerning, considering this generation’s widespread use of the internet as a source of information. The findings of this study have provided baseline data of the health literacy of Malaysians and provide evidence suggesting potential areas of intervention.

Compared to global trends, some of the findings of this study offered different views on the relationships between socio-demographic factors and health literacy. This shows that the association between health literacy and socio-demographic factors may not conform to a singular pattern. The investigation of different contexts and different populations should be encouraged in order to enrich our understanding of global health literacy.

## Figures and Tables

**Table 1:** Sample distribution.

State	Locality	Ethnicity	N	Area
Peninsular—Selangor (6.298 mil = 58.2%) *n*= 466	Urban (93.3%) *n* = 435	Malay	299	Shah Alam
		Chinese	103	
		Indian	33	
	Rural (6.7%) *n* = 31	Malay	21	Hulu Langat
		Chinese	7	
		Indian	3	
Peninsular—Kuala Lumpur (1.782 mil = 16.5%) *n* = 132	Urban (100%) *n* = 132	Malay	82	Segambut and Lembah Pantai
		Chinese	38	
		Indian	12	
Borneo—Sarawak (2.741 mil = 25.3%) *n*= 202	Urban (57.8%) *n* = 117	Bumiputera	91	Kuching
		Chinese	26	
		Indian	0	
	Rural (42.2%) *n* = 85	Bumiputera	66	Sarikei (Maradong) and Samarahan (Simujan)
		Chinese	19	
		Indian	0	

Note: Location of study is determined by population density and ethnic distribution.

**Table 2:** Distribution of respondent characteristics and health literacy levels using HLS-M-Q18 (*N* = 866).

	*N* (%)	Health Literacy Level *N* (%)			
		Inadequate	Problematic	Sufficient	Excellent
**Respondents**	866 (100)	154 (17.8)	348 (40.2)	284 (32.8)	79 (9.1)
**Age** (mean)	866 (33.6)	33.1	33.8	33.5	33.8
Gen Z (1995–2012)	211 (24.4)	33 (15.6)	75 (35.5)	78 (37.0)	25 (11.8)
Gen Y (1977–1994)	377 (43.6)	73 (19.4)	162 (43.0)	114 (30.2)	28 (7.4)
Gen X (1966–1976)	184 (21.3)	34 (18.5)	77 (41.8)	58 (31.5)	15 (8.2)
Baby Boomers (1950–1965)	93 (9.8)	13 (14.0)	34 (36.6)	35 (37.6)	11 (11.8)
**Gender**					
Male	303 (35)	65 (21.5)	109 (36.0)	105 (34.7)	24 (7.9)
Female	563 (65)	89 (15.8)	239 (42.5)	180 (32.0)	55 (9.8)
**Race**					
Malay	470 (54.3)	68 (14.5)	188 (40.0)	170 (36.2)	44 (9.4)
Chinese	213 (24.6)	46 (21.6)	89 (41.8)	59 (27.7)	19 (8.9)
Indian	65 (7.5)	15 (23.1)	21 (32.3)	23 (35.4)	6 (9.2)
Bumiputera	115 (13.3)	25 (21.7)	48 (41.7)	32 (27.8)	10 (8.7)
**Marital status**					
Not married	429 (49.7)	74 (17.2)	163 (38.0)	149 (34.7)	43 (10.0)
Married	394 (45.6)	74 (18.8)	162 (41.1)	125 (31.7)	33 (8.4)
Separated/Divorced	21 (2.4)	4 (19.0)	12 (57.1)	4 (19.0)	1 (4.8)
Widowed	20 (2.3)	2 (10.0)	10 (50.0)	6 (30.0)	2 (10.0)
**Income**					
Below RM3000 (including no income)	510 (59.4)	88 (17.3)	220 (43.1)	162 (31.8)	40 (7.8)
RM3001–RM9000	293 (34.1)	58 (19.8)	105 (35.8)	99 (33.8)	31 (10.6)
≥RM9001	55 (6.4)	7 (12.7)	20 (36.4)	20 (36.4)	8 (14.5)
**Exercise (days a week)**					
0–2 days	347(40.1)	85 (24.5)	130 (37.5)	111 (32.0)	21 (6.1)
More than 2 days a week	519 (59.9)	69 (13.3)	218 (42.0)	174 (33.5)	58 (11.2)
**Health Problems**					
1 and more than 1 disease	219 (25.3)	42 (19.2)	84 (38.4)	76 (34.7)	17 (7.8)
No disease	646 (74.7)	17.3 (17.3)	40.9 (40.9)	32.2 (32.2)	9.6 (9.6)
**Health status**					
Bad	248 (28.7)	70 (28.2)	105 (42.3)	61 (24.6)	12 (4.8)
Good	617 (71.3)	84 (13.6)	242 (39.2)	224 (36.3)	67(10.9)

**Table 3:** Odds ratios (95% confidence intervals) of having limited health literacy vs. adequate health literacy (*N* = 866).

	Adequate Health Literacy (Yes = 1)			
	*p* Value	Exp (B)	95% C.I for Exp (B)	
			Lower	Upper
^a^ Gender—Male	0.843	0.969	0.709	1.324
^b^ Age				
Gen Z	0.478	0.793	0.417	1.505
Gen Y	0.031	0.549	0.319	0.946
Gen X	0.179	0.682	0.390	1.191
^c^ Race				
Chinese	0.115	0.751	0.526	1.073
Indian	0.886	0.960	0.553	1.667
Bumiputera	0.177	0.735	0.470	1.149
Others	0.724	0.638	0.053	7.720
^d^ Health Status				
Bad	0.000	0.431	0.301	0.618
^e^ Health Problem				
One or more than one	0.050	1.447	1.000	2.096
^f^ Marital status				
Not married	1.000			
Married	0.269	0.814	0.565	1.173
Separated/divorced	0.076	0.381	0.131	1.105
Widowed	0.383	0.631	0.224	1.775
^g^ Income				
Below RM3000 (including no income)				
RM3001–9000	0.122	1.281	0.936	1.754
≥RM9001	0.126	1.580	0.879	2.841
^h^ Exercise (days a week)				
More than 2 days a week	0.252	1.186	0.885	1.590

^a^ Female respondents used as a reference. ^b^ Respondents aged > 60 years (Baby Boomers) used as a reference. ^c^ Respondents who stated their ethnicity as “Malay” used as a reference. ^d^ Respondents who stated their health status as “Good” used as a reference. ^e^ Respondents who stated their health problem as “No disease” used as a reference. ^f^ Respondents who stated their marital status as “Not married” used as a reference. ^g^ Respondents whose income was below RM3000/month used as a reference. ^h^ Respondents who stated their daily exercise as “≤2 days a week” used as a reference.

## Data Availability

The dataset for this study is available upon request from the funder, The Institute for Health Behavioural Research, Ministry of Health, Malaysia.

## Supplementary Materials

The following are available online at https://www.mdpi.com/article/10.3390/ijerph18094860/s1, Table S1: Research Dataset.
